# A spinal network of proprioceptive reflexes can produce a variety of bipedal gaits

**DOI:** 10.1038/s42003-025-09307-x

**Published:** 2025-12-16

**Authors:** Elsa K. Bunz, Daniel F. B. Haeufle, Syn Schmitt, Thomas Geijtenbeek

**Affiliations:** 1 Institute for Modelling and Simulation of Biomechanical Systems, University of Stuttgart, Stuttgart, Germany; 2 Stuttgart Center for Simulation Science, University of Stuttgart, Stuttgart, Germany; 3 Hertie Institute for Clinical Brain Research and Center for Integrative Neuroscience, Tuebingen, Germany; 4 Center for Bionic Intelligence Tuebingen Stuttgart, Tuebingen Stuttgart, Stuttgart, Germany; 5 Goatstream, Utrecht, The Netherlands

**Keywords:** Computational biophysics, Sensory systems, Musculoskeletal system, Biophysical models, Computational models

## Abstract

Proprioception is crucial for movement, yet the role of proprioceptive reflexes in legged locomotion is still poorly understood. While previous simulation studies have shown great potential for reflex-based control strategies, these controllers are typically geared to specific gaits, using hand-crafted feedback pathways that are linked to specific gait phases. In this work, we explore the control capabilities of a simple reflex controller that consists of only homonymous and antagonistic length and force feedback pathways with constant gains. This control model can be considered a highly simplified subset of spinal control rather than an attempt to emulate all spinal control functions. Despite its simplicity, we found our control framework capable of producing a wide variety of natural gaits, including walking and hopping, forwards and backwards, and running in different variations and at different velocities – without requiring any rhythmic inputs or high-level state machines modulating the feedback gains. Our work highlights the important role and flexibility of proprioceptive reflexes and suggests a necessary re-evaluation of their role in locomotion. Due to its simplicity and flexibility, our control framework provides a solid basis for the development of higher-level neuromuscular control systems.

## Abstract (web summary)

A spinal reflex controller with fixed-gain muscle force and length feedback can generate a wide range of natural gaits, offering insight into how proprioceptive reflexes may contribute to locomotor control.

## Introduction

The role of proprioceptive reflexes in human and animal locomotion has been subject to long-standing research and debate^1,2^. In 1906, after observing that decerebrated cats could still produce basic stepping movements, Charles Sherrington proposed that complex movements arise from sequential reflex chains linking sensory input to motor output^3–5^. In 1911, Thomas Graham Brown discovered that rhythmic locomotor activity persisted even after removing sensory input to the spinal cord, indicating the presence of intrinsic spinal circuits—later dubbed *central pattern generators* or CPGs—capable of producing rhythmic motor output without requiring sensory input signals^2,6,7^. Neuroscientific research often highlights the importance of CPGs^8–10^, yet their exact functioning in the human spinal cord remains unclear^2^. Information from proprioceptive sensors has been shown to be essential for movement generation^11^, the regulation of stepping^12^, and the modulation of CPG output^13,14^. However, the way in which CPGs and reflexes functionally interact remains poorly understood, and in vivo preparations are insufficient to decipher the parameters and structures involved in this interaction, particularly during dynamic locomotion^8,15^.

Predictive neuromuscular simulations are a useful alternative to in vivo preparations, since they allow complete and fine-grained control over the experimental setup and help identify the potential role of individual control primitives^1,16^. In addition, predictive neuromuscular simulations have important potential applications, such as aiding in the design of assistive devices through human-in-the-loop simulation^17–21^, predicting the outcome of medical procedures^22–24^, and predicting gait stability and perturbation responses^25–27^. However, to fulfill these promises, a control framework is needed that is both neurologically grounded and capable of replicating the versatility of human movement.

An important simulation study that highlights the potential of reflexes in locomotion is the work of Geyer and Herr^28^, who developed a locomotion control strategy primarily driven by proprioceptive reflexes. Their work inspired a large number of derivative controllers, including 3D extensions and the addition of higher-level control components^29–33^ and was the basis for several subsequent predictive simulation studies on human gait^34–36^. These controllers produce rhythmic muscle activation by switching between specific reflex pathways based on the current state of each leg (i.e., swing and stance), using hand-crafted state selection logic. Furthermore, reflex pathways are domain-specific, as they are manually picked to mimic human-level ground walking kinematics and muscular activations. This tailoring to level-ground walking and dependence on state-switching limit their ability to generalize to other gaits. Work extending the controller of Geyer and Herr^28^ to other environments or movements mainly focuses on adding a higher-level component^25,30^ (Song and Geyer^29^ also add new reflex pathways in addition to a supraspinal layer) or on further refining and tailoring the states used^32,33^. While this work has provided insights on different parts of the human motor control system, this approach may obscure the potential within reflexes themselves. An important avenue of future research is to extend the capability of these control systems and allow for more complex tasks^37^, which requires a solid spinal foundation.

**Fig. 1:**
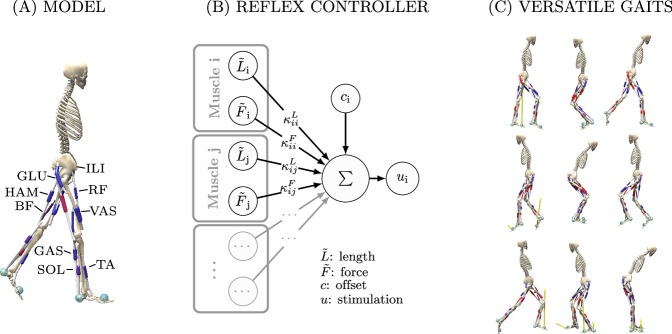
Overview. Our reflex controller can produce a variety of gaits in a human musculoskeletal model. **A** The planar musculoskeletal model consists of seven segments connected by 6 joints, which are actuated by 18 Hill-type muscles. **B** Our reflex controller calculates stimulation for each muscle *i* from delayed force $\tilde{F}$ and length $\tilde{L}$ feedback with gains *κ*_*ij*_ and a constant offset *c*_*i*_. Implemented pathways include homonymous and antagonistic pathways for length and force. Feedback gains and offsets are optimized based on high-level objectives. **C** The controller can produce a variety of bipedal gaits like forwards and backwards walking, and hopping as well as running.

The purpose of this study is to explore the locomotor capabilities of proprioceptive reflexes in the absence of a high-level state selection or modulation. Instead, we use delayed length and force reflex pathways with constant feedback gains, which are active throughout the simulation, relying solely on reciprocal innervation to produce alternating rhythmic activity (see Fig. 1). For reflex connectivity, our architecture is informed by the basic organizational principles of the mammalian spinal cord^13^ and includes reflex pathways to homonymous and antagonistic muscles. For the sake of simplicity, we have opted to exclude other sensory input modalities, such as muscle fiber velocity, cutaneous sensors, and vestibular sensors. As such, our control model can be considered a highly simplified subset of spinal control rather than a full emulation. Preliminary studies have shown that adding these input modalities did not result in noticeable improvement in the discovered gait patterns.

In spite of its simplicity, we found that these basic reflexes were sufficient to produce a variety of natural bipedal gaits like walking, hopping, and running in different variations and at different velocities—only by selecting different sets of constant feedback parameters. With its versatile functionality, it can be considered a basic starting point on top of which more elaborate control strategies can be developed.

## Results

For all five target gaits, we found solutions that did not fall within the maximum simulation time and enter rhythmic cycles (see Video S1 and phase plots Fig. S6). The model reaches the target velocity of 1.2 m/s for forwards and backwards walking and a maximum running velocity of 3.4 m/s. The hopping solutions generate hopping forwards and backwards at a velocity of 1.2 m/s and 1.4 m/s, respectively (compare also Table 1).

**Table 1:** Gait velocity

	Walk Fwd	Walk Bkw	Hop Fwd	Hop Bkw	Run
Velocity (m/s)	1.20	−1.21	1.22	−1.39	3.42
Stride length (m)	1.11	−0.83	0.54	−0.54	1.94
Stride duration (s)	0.92	0.69	0.44	0.38	0.57

Obtained velocities, stride length, and duration for the five target gaits.

**Fig. 2:**
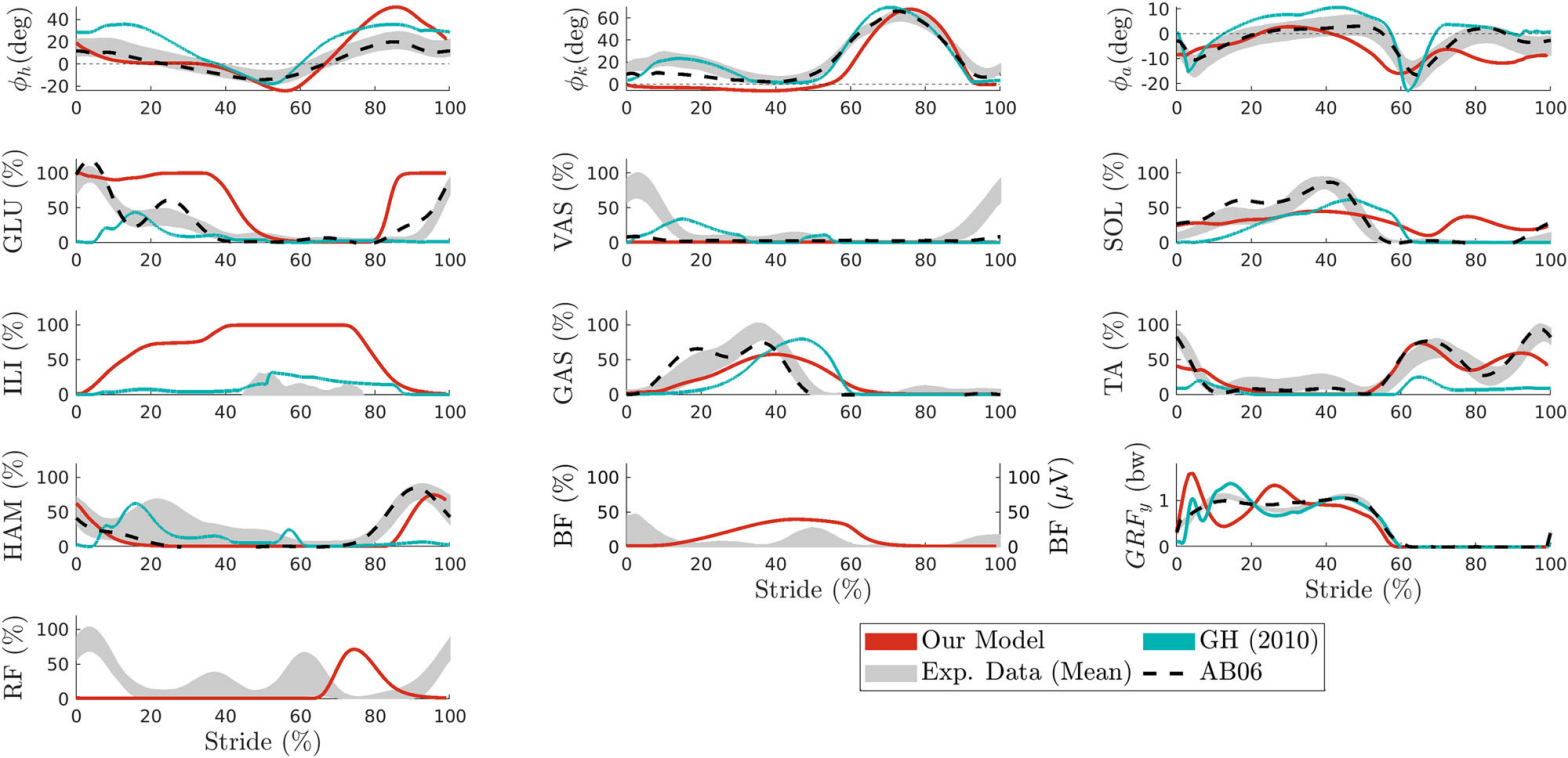
Forwards walking. Joint kinematics (first row) and corresponding muscular activation for hip (left), knee (middle), and ankle (right) joints, as well as ground reaction force (bottom right). Data are shown for the mean of all strides of our forward walking solution (red) compared to the model of Geyer and Herr^28^ (blue), experimental data averaged over all subjects from refs. ^38,76,77^ (gray area, see also Section “Experimental data''), and of one height and weight matched subject AB06 from Camargo et al.^38^ (dotted black).

Our forwards walking gait matches human kinematics well (see Fig. 2, *ϕ*_*h*, *our*_: *R* = 0.90, *ϕ*_*k*, *our*_: *R* = 0.89, *ϕ*_*a*, *our*_: *R* = 0.79). Even though the knee kinematics are not matched as well as by the reflex controller of Geyer and Herr^28^(*ϕ*_*k*, *GH*_: *R* = 0.98), the ankle kinematics show a greater maximum cross-correlation ^28^(*ϕ*_*a*, *GH*_: *R* = 0.69). The mismatch in knee kinematics is mostly coming from the leg being straight during stance. The vertical ground reaction force shows the characteristic two peaks found in experimental data (albeit with shifted timings, *R* = 0.93). Generally, the computed time shifts Δ of the maximum cross-correlation for knee, ankle, and GRF are higher for our model than for the model of Geyer and Herr^28^, but within maximally 3.6% (Δ_*GRF*_). All maximum cross-correlation values and time shifts are given in Table 2.

**Table 2:** Maximum cross-correlation *R* and time shift *Δ* for joint angles and GRF

	*ϕ*_*h*_		*ϕ*_*k*_		*ϕ*_*a*_		GRF	
Walk Fwd	*R*	*Δ* (%)	*R*	*Δ* (%)	*R*	*Δ* (%)	*R*	*Δ* (%)
exp-our	0.90	0.0	0.89	2.1	0.79	3.4	0.93	3.6
exp-GH	0.93	0.0	0.98	0.6	0.69	1.0	0.99	0.7
AB06-our	0.91	0.0	0.95	2.8	0.72	4.1	0.94	2.7
AB06-GH	0.81	0.0	0.98	0.3	0.75	1.7	0.98	0.7

Run								
exp-our	0.91	0.0	0.93	2.0	0.25	20	0.78	8.5

Similarity metrics computed between the mean of experimental data (exp) or mean data of subject AB06 (for Walk Fwd only) and simulation data from our model (our) or the model of Geyer and Herr^28^ (GH, Walk Fwd only).

Also, in terms of muscular activations, the controller produces smooth muscular activations that match experimental data reasonably well. Maximum cross-correlation values can be found in Table S3. The straight knee during stance is caused by an absence of VAS activation. Among the variations of forwards walking we found, there is also one gait with a knee flexion during stance and VAS activation, as well as less shifted ground reaction forces (see Fig. S7b for kinematics and activations and Tables S3 and S4 for maximum cross-correlation results). Therefore, this mismatch is likely a result of either the lack of reflex modulation that makes it difficult to differentiate between stance and swing, or the generic cost function, or a combination of both. Note, however, that the gait is still close to human walking as humans exhibit a wide variety of gait kinematics and muscular activations (e.g., height and weight matched subject AB06 from Camargo et al.^38^ (see Fig. 2 and Table 2) also shows very little VAS activation and a relatively straight leg during stance, *ϕ*_*k*, *AB*06−*our*_: *R* = 0.95).

**Fig. 3:**
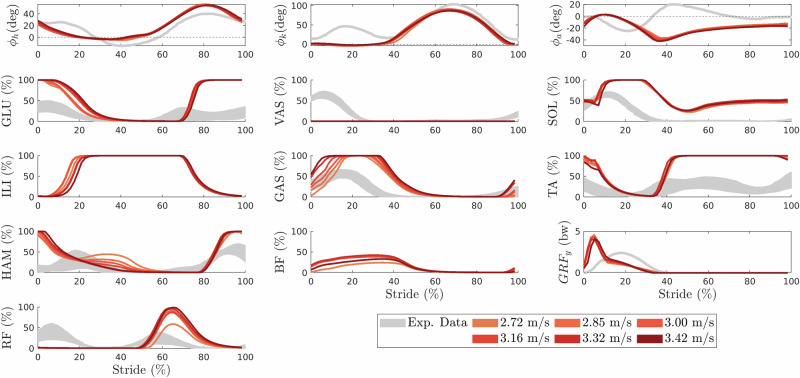
Running. Joint kinematics (first row) and corresponding muscular activations for hip (left), knee (middle), and ankle (right) joints, as well as ground reaction force (bottom right) for running at different velocities in comparison to experimental data of running at 3.0 m/s from Hamner and Delp^79^ (gray).

For our running targets, we discovered various solutions with different running velocities. Figure 3 shows the kinematics and muscular activations for running gaits at different velocities ranging from 2.72 to 3.42 m/s. While ankle kinematics are not accurately matched (*ϕ*_*a*_: *R* = 0.25, Δ = 10%), hip and knee angle match experimental data well (*ϕ*_*h*_: *R* = 0.91, *ϕ*_*k*_: *R* = 0.93), even though, as in Walk Fwd, the knee is too straight during stance. Also in Run, ground reaction forces are shifted, and initially ground reaction forces are substantially higher in the model than in the experimental data (*R* = 0.78, Δ = 8.5%). Except for VAS, which is not activated at all and RF which misses activation during stance, muscular activations show a fair agreement with experimental data (0.8 < *R* < 0.91, see Table S5), but some muscles saturate at maximum activation (GLU, SOL, ILI, GAS, TA) probably because only speed was maximized and effort was not taken into account.

The kinematics and muscle activations for Walk Bkw, Hop Fwd, and Hop Bkw are provided in Fig. S10. In an iterative, hand-tuning process, we were furthermore able to find a wide spectrum of solutions for each target gait (see Video S2). Also, a variety of other related gaits, including skipping or backwards running, can be generated. To illustrate the versatility of the reflex controller, we compiled a collection of some other interesting gaits we found (see Video S3). While these gaits are often suboptimal, they still demonstrate a lively appearance that is difficult to quantify.

**Fig. 4:**
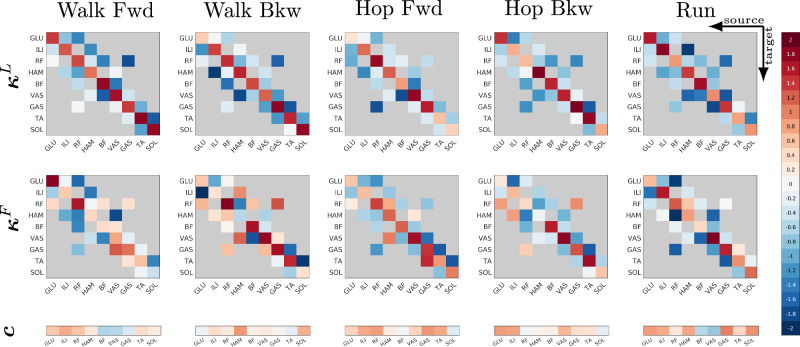
Controller parameters. Parameter values for the five target gaits (left to right) for length (top row) and force (middle row) reflexes, as well as the constant prestimulations (bottom row). Matrix columns represent the source muscle the feedback is coming from, rows show the target muscle receiving a stimulation input from the reflex connection.

A comparison of the parameter values of the five target gaits provides some insights into the parameter space of the controller (see Fig. 4). Generally, the solutions are very diverse and differ a lot between the different gaits (see Fig. S1) as well as within gait (see Fig. S8 for parameter values of Walk Fwd 2, a second, manually optimized solution for Walk Fwd). Directly interpreting and comparing prominent reflexes is difficult, as the interference between reflexes can cancel out the influence of a reflex such that its value becomes meaningless (e.g., a large negative prestimulation will cause an inactive muscle even if other reflexes would generate an activation of this muscle).

However, more generally, we find that the offsets *c*_*i*_ are higher for the gaits without effort minimization (hopping and running) than for the walking gaits, where effort minimization was included. Also, homonymous feedback was not only excitatory for the length reflexes, but also for most of the force reflexes, even though this was not a constraint during the optimization. This supports the idea that positive homonymous force feedback is important for human gait^39,40^. Overall, we find that for all five target gaits, most reflexes are active, with only a few reflex gains set to an absolute value below 0.1 (Walk Fwd: 3, Walk Bkw: 5, Hop Fwd: 1, Hop Bkw: 4, Run: 2). However, as argued before and as the example of VAS during walking and running shows, not all of these reflexes have an influence as they might get canceled out by prestimulations or other reflexes. In fact, the additional walking solution Walk Fwd 2 generates forwards walking with overall smaller absolute reflex gains and 19 reflexes below 0.1 (compare also Fig. S8).

## Discussion

Our results demonstrate that a simple set of reflexes with constant feedback gains is enough to produce a wide variety of bipedal gaits—without the need for a state-switching mechanism or central pattern generator. Since our controller does not depend on hand-picked connections or finite-state machines specifically tailored to walking, it can generalize to other natural-looking gaits, exposing the remarkable potential of reflex-based control. We consider the natural versatility in the absence of high-level control to be the main strength of our controller.

The absence of a state switch mechanism and the restriction to homonymous and antagonistic length and force feedback are the main differences between our control architecture and the controller of Geyer and Herr^28^. However, due to its reflexive nature, our controller shares many features with the controller of Geyer and Herr^28^ and subsequent works^29,33^. First, inter-stride variability is normally relatively low as the periodic movement is based on an interplay of sensor signals (details see also Supplementary Material Section 3). Second, the initial state of the model is important and set close to the target motion as the reflex controller is dependent on the sensory feedback to get into equilibrium. In nature, supraspinal mechanisms could drive the system to the specific initial condition where reflexes can take over completely. Third, even though these controllers are not good at tracking kinematics, desired trajectories can be used in the cost function to discover reflex parameters that lead to similar kinematics. On the other hand, kinematics emerge naturally from an interplay between body mechanics, muscles, and control, and generally, muscular activations are smooth and natural without abrupt changes.

Our controller has the ability to naturally converge to human-like activations and walking kinematics, without the use of experimental data during the optimization. Kinematically, the main difference to experimental data is the straight knee during stance. The controller proposed by Geyer and Herr^28^ better reproduces knee angle and ground reaction forces. We suspect that modulation of the VAS reflexes (which Geyer and Herr^28^ approximated through state-switching) is important to properly differentiate between passive VAS during swing and active VAS during stance.

In terms of muscular activation, our results are similar to the ones of Geyer and Herr^28^: for some muscles, our controller better tracks the experimental data, while for others, their controller is more accurate, and both models do show differences to experimental data. Generally, experimental studies show there is a wide range of muscle activity patterns both intra-subject between strides as well as inter-subjects^41,42^. Our controller captures part of this diversity and allows for different forwards walking solutions with different kinematics (e.g., knee flexion during stance), but also differences in muscle activation patterns of several muscles (GLU, VAS, HAM, BF for Walk Fwd compared to Walk Fwd 2). The full spectrum of gait variability of our reflex controller may still be further explored.

Our controller generalizes better to different gaits than the work of Geyer and Herr^28^, which has been tailored to level-ground walking and therefore is not easily extended to other gaits. Song and Geyer^29^ impressively extended the controller to allow for more complex movements like turning movements and running, which required not only additional reflexes and states, but also a supraspinal control layer. They also found the trunk lean to be an important variable to modulate running speed^43^. Our controller can generate running at different speeds (and backwards walking and hopping) without any change to the controller architecture, using only length and force reflexes. The maximum velocity of 3.42 m/s is well below the maximum velocity professional athletes can run at (average velocity for world record for marathon: 5.8 m/s or 5000 m: 6.62 m/s^44^), however, it is close to the mean preferred running speed of 3.7 m/s found in experienced runners^45^. Also, an increase of maximal muscle forces (mimicking training) could likely increase the maximum velocity of our model. We also expect the simplicity of the model and lack of arms to contribute to the slower maximum speed^46^.

All presented results are the starting point for further development and studies to overcome its current limitations. We adopted the current approach to allow studying the broad versatility of the control structure. When focusing on one specific gait, the differences to experimental data could possibly be minimized by a more complex cost function including further optimization criteria like joint pain or kinematic tracking. Kinematic tracking could also provide a way to model subject differences and analyze differences in the resulting reflexes. Even though it might seem counter-intuitive in a forward simulation, for reflex controllers, this provides a neat way to constrain the kinematics, as the reflex controller can not directly follow a trajectory and therefore the kinematics only shape the cost function.

In the same line of studying the broad versatility of the controller, we allowed for all homonymous and antagonistic reflexes, such that the potential of the controller could be explored, and no bias was introduced by selecting reflexes more specifically. This, however, limits the analysis and interpretability of the found solutions, as likely not all reflexes are needed for all gaits, and interaction effects between parameters can lead to reflexes without effect, even if the reflex gain is high. In the future, these limitations could be overcome by a detailed study of the parameter space, including a minimization of active reflexes, an analysis of the connections within as well as between gaits, and an assessment of the initial state dependency. With this, the dependency on the cost function and hyperparameters could be eliminated, providing a more continuous view on the parameter space. A minimization of reflexes could help to obtain a sparser controller structure and a more meaningful set of reflexes that can be better compared to neurophysiological data, and reduce the number of redundant or unnecessary reflexes. The focus of this work was to showcase the versatility and potential of the control structure; however, the additional solution for forwards walking we found by manual tuning and iterative optimization already demonstrates that potentially less reflexes are necessary and might lead to more natural-looking results.

We purposefully designed our controller to be as simple as possible, only including a minimal set of components and sensor signals (specifically, muscle fiber length and force). We recognize that the absence of velocity feedback may appear problematic, but results from our preliminary simulations indicated that including velocity feedback did not result in noticeable improvement of the discovered gait patterns. We believe that further study is required to understand why the effect of including velocity feedback appears limited, compared to using only length feedback from muscle spindles. One hypothesis worth exploring is that additional filtering of the velocity signal is needed, since the velocity of the contractile element in simulated Hill-type muscles can fluctuate rapidly, potentially limiting its suitability as a direct feedback signal.

Our finding that this minimal network of length and force feedback can produce a rich variety of periodic gaits is surprising and encouraging. It is, thus, a functional and versatile subset of spinal control. Still, it deliberately excludes mechanisms shown elsewhere to enhance reflexive adaptability: Rybak et al.^47^ described spinal CPG architectures where multimodal feedback (length + velocity + phase-specific inputs) enhances phase transitions and robustness in cat models. Loeb^48^ and also Prochazka and Gorassini^49^ emphasized how fusimotor drive modulates spindle gain across task conditions. Likewise, recent works have demonstrated that *γ*-fusimotor modulation nonlinearly shapes spindle output and that anatomical agonist-antagonist definitions may be more functionally fluid than our current rigid reciprocal inhibition network^50,51^. Incorporating these features—velocity feedback, dynamic fusimotor control, and flexible reciprocal inhibition—could significantly improve adaptability, perturbation resistance, and context-sensitive gait modulation.

Our controller can easily be extended in many ways. The inclusion of supraspinal mechanisms to control gait initiation, speed, or transitions is an important direction for future research. This could also solve the dependency on the initial state, as supraspinal mechanisms could take over until a state is reached that allows for fully reflexive control. Also, a change of gait means a complete change of parameters in our current architecture, which is not plausible in humans, even though reflexes do change, e.g., between postural control and locomotion^52^. The proposed controller provides a valuable basis for extensions to more complex and targeted, voluntary movements, as well as different terrains and perturbations demanding a higher-level control structure.

Overall, our proposed methodology is sufficient to demonstrate the power of the spinal reflex control approach without high-level state selection or modulation. To our knowledge, this is the first controller that can generate different gaits using only reflexes and no higher-level control structure, finite-state machine, or CPG. It provides a solid basis for examining the relation between different gaits and incrementally developing more complex neuromuscular controllers^37^.

Reflex controllers have also shown potential in the control of exoskeletons, prosthesis, and robots, as they allow for more realistic human-in-the-loop simulation^20,21,53^. Because our controller does not rely on gait state detection, it may be easier to incorporate in these scenarios.

## Conclusion

Our results demonstrate that proprioceptive reflexes are a remarkably powerful control primitive and that state-switching with a finite state machine or a CPG is not needed to generate rhythmic bipedal gaits. Furthermore, our reflex controller allows for a striking versatility of gaits, suggesting that spinal reflexes play a more prominent role in gait than expected. Even though we know that other circuits, such as central pattern generators, play an important role in gait, their importance, relation, and dependencies might need to be re-evaluated. Our proposed controller provides a valuable basis to develop more complex neuromuscular controllers and has possible applications in the control of robots, exoskeletons, prostheses, or ortheses.

## Methods

### Model

Simulations were performed using a planar musculoskeletal model representing an adult human male of 74.5 kg and 1.8 m height. The model consists of 7 segments (torso and two legs with femur, tibia, and foot) and 9 degrees-of-freedom (DOFs). Hip, knee and ankle joint of each leg are actuated by 9 Hill-type musculotendon units with elastic tendons and muscle fiber damping^54^: gluteus (GLU), iliopsoas (ILI), rectus femoris (RF), hamstrings (HAM), biceps femoris short head (BF), vastus (VAS), gastrocnemius (GAS), tibialis anterior (TA), and soleus (SOL) (see also Fig. 1). Both the mass properties and muscle parameters in our model (optimal fiber length, tendon slack length, pennation at optimal fiber length, maximum isometric force) were taken from Delp et al.^55^, with the exception of the hamstring parameters, for which we used updated data from Arnold et al.^56^. We further adapted the via points of the muscle pathways to match moment arm data from Rajagopal et al.^57^, which uses wrapping geometry to match experimentally measured moment arms. We avoided the use of wrapping geometry in our model for performance reasons, but found that through via-points we could match the moment arms within the margin of error of the source material.

In cases where we changed the muscle geometry, we adjusted the tendon slack lengths in such a way that the muscle fiber lengths remained optimal at the same joint angle (or joint angles for biarticular muscles), thereby minimizing changes in the angle at which passive muscle force commences. The latter is important because our control strategy relies on force feedback, which is sensitive to the passive muscle force that is triggered after a muscle stretches past its optimal fiber length. Following that same rationale, we adjusted the tendon slack length of the ankle plantarflexors in such a way that these muscles better match passive dorsiflexion muscle force measurements found in Le Sant et al.^58^. The curves representing tendon stiffness, as well as the force-length and force-velocity relations, are taken from Millard et al.^54^, including the passive damping (*β* = 0.1), allowing muscle activation levels to be zero. We use a *v*_max_ of 10 m/s, similar to Delp et al.^55^ and Rajagopal et al.^57^. Muscle activation is computed using a first-order dynamics model, with an activation time constant of 0.01 s and a deactivation time constant of 0.04 s^59^.

Ground contacts are modeled using the Hunt-Crossley contact model^60^ with two contact spheres (*r* = 0.03, stiffness = 5 × 10^6^, dissipation = 1) per foot segment, using the friction model implemented in OpenSim^61^ (static friction = 0.9, dynamic and viscous friction = 0.6). The model is implemented in SCONE^62^ and Hyfydy^63^ and is provided as supplementary material. An overview of the model parameters is provided in Table S1.

### Control

Our neural control model is based on proprioceptive feedback from muscle spindles (type Ia and II sensory fibers) and Golgi tendon organs (type Ib sensory fibers)^13,64^. Our aim is not to replicate the full neurophysiological complexity of the spinal cord or to model a full decerebrated preparation, but rather to capture only a minimally functional subset. In our model, muscle spindle receptors signal information about muscle fiber length, and Golgi tendon organ receptors signal information about muscle tension. Even though Ia feedback from muscle spindles includes both fiber length and velocity information, we were unable to see the benefits of including velocity feedback in the results of our preliminary studies (see Discussion for more details). To strike a balance between controller simplicity and capability, and to keep the number of optimization parameters to a minimum, we therefore chose to leave out fiber velocity feedback and focus only on length and force feedback. The investigation of other sensory input modalities, which are also known to be important for human gait^65,66^, is left for future work.

Our model incorporates the following simplified reflex pathways commonly observed in the mammalian spinal cord: homonymous reflex connections to the same muscle and antagonistic reflex connections to all antagonist muscles that cross the same joint^11,13,67^. For length reflexes, homonymous connections are considered monosynaptic and constrained to be excitatory, while antagonistic connections are constrained to be inhibitory, representing connections via Ia inhibitory interneurons. Ib force reflexes are allowed to be either excitatory or inhibitory. While it is well known that Ib afferents have a widespread inhibitory influence, it has been observed directly in cats that homonymous Ib afferents can initiate excitatory responses during locomotor activity^68^, which has been suggested to play an important role in human gait as well^39,40^.

**Fig. 5:**
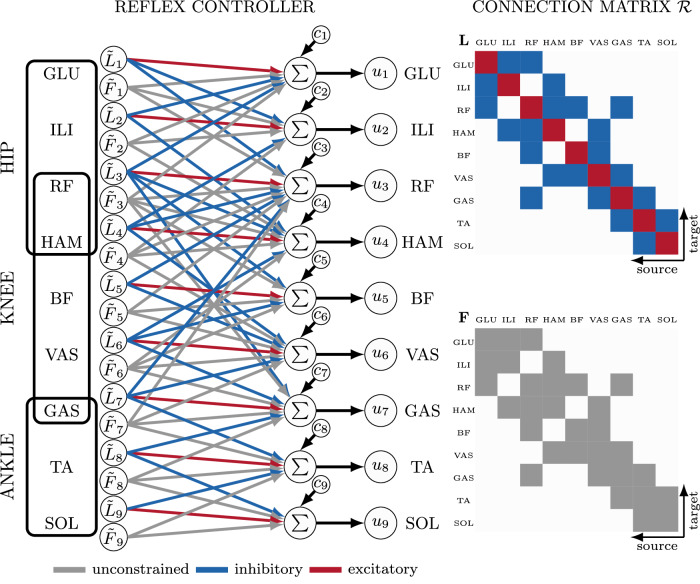
Control architecture. Muscle stimulations *u*_*i*_(*t*) for the nine muscles per leg are computed through delayed length ($\tilde{L}(t-\Delta t)$) and force ($\tilde{F}(t-\Delta t)$) feedback. The connection matrix ${{{\mathcal{R}}}}$ (right) contains both homonymous connections innervating the muscle based on its own sensor signal (red in *L* matrix) and connections between antagonistic muscles (blue in *L* matrix). Gains are either unconstrained (gray, ${\kappa }_{ii}^{F}$ and ${\kappa }_{ij}^{F}$), constrained to be excitatory (i.e., ≥0, red, ${\kappa }_{ii}^{L}$) or inhibitory (i.e., ≤0, blue, ${\kappa }_{ij}^{L}$). Including the constant thresholds *c*_i_, the reflex controller contains 71 free parameters.

For our model with 9 muscles per leg this leads to connection matrix ${{{\mathcal{R}}}}$ containing 9 monosynaptic (red, Fig. 5) and 22 antagonistic connections (blue, Fig. 5). All pathways are modeled with neural delays Δ*t* ∈ [10 ms, 35 ms] based on experimental data of H-reflex latency as well as the length of the pathway (i.e., the more distal the more delay), following van der Kruk and Geijtenbeek^69^ (for the delay values see Table S1).

Overall, the controller thus produces a stimulation *u*(*t*) of each muscle *i* based on constant-gain delayed length ($\tilde{L}$) and force ($\tilde{F}$) feedback, and a constant offset *c*_*i*_:

1 $${u}_{i}(t)={c}_{i}+{\sum }_{j\in {{{{\mathcal{R}}}}}_{i}}^{}[{\kappa \, }_{ij}^{L}{\tilde{L}}_{j}(t-\Delta t)+{\kappa \, }_{ij}^{F}{\tilde{F}}_{j}(t-\Delta t)]$$

Here, *u*_*i*_ is saturated to [0,1], ∣*κ*∣ ≤ 2, ∣*c*∣ ≤ 1. We use the same pathways and control parameters (*κ* and *c*) for both legs. Our controller does not include cross-connections between the left and right legs. The normalized muscle fiber length $\tilde{L}$ and force $\tilde{F}$ are defined as:

2 $${\tilde{L}}_{i}(t)=\frac{{L}_{i}(t)}{{l}_{{\mbox{opt}},i}}-{l}_{0}{{\mbox{and}}}\,{\tilde{F}}_{i}(t)=\frac{{F}_{i}(t)}{{F}_{{\mbox{max}},i}}$$

with *l*_opt,*i*_ the optimal fiber length and *F*_max,*i*_ the maximal isometric force of muscle *i* (see Table S1). We set *l*_0_ = 0.5 to ensure ${\tilde{L}}_{i}(t)$ is within a realistic range while avoiding length inputs to become negative. All reflex gains and offsets remain constant for the duration of the simulation; there is no modulation or switching based on different states. Since the offsets represented by *c*_*i*_ also remain constant, they do not constitute a feedforward control signal and are functionally identical to the length offsets represented by *l*_0_.

### Optimization

Our reflex controller contains 71 free parameters (9 *c*_*i*_, 31 ${\kappa }_{ij}^{L}$ and 31 ${\kappa }_{ij}^{F}$), which dictate the behavior produced by the controller. We explore the capabilities of the reflex controller by defining five target gaits: forward walking (Walk Fwd) and hopping (Hop Fwd), backwards walking (Walk Bkw) and hopping (Hop Bkw), and running (Run).

**Fig. 6:**
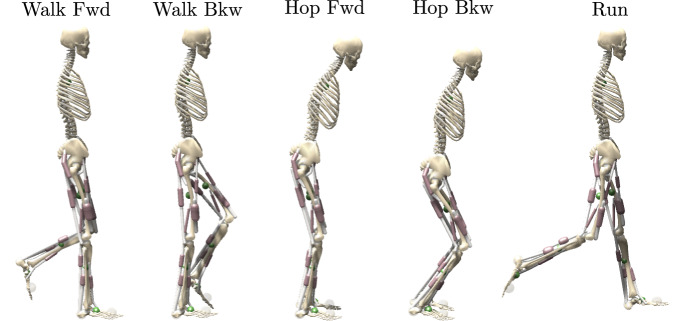
Initial states. For each target gait, the simulation starts in a different initial pose (joint angles and velocities). These poses are manually tuned to minimize the initial correction the controller has to make and to direct the optimization towards the target gait. The numeric values of all initial sets can be found in Table S2.

For the five target gaits, based on the initial gaits we found, we extracted and manually tuned the initial state of the model (torso and joint angles and velocities) to roughly reflect the target gait (see Fig. 6 and Table S2). To produce a periodic motion, the reflex controller needs to find an equilibrium where the interplay of reflexes produces cyclically reoccurring states and sensor signals. At the beginning of the simulation, the system first has to initialize the feedback signals and enter the limit cycle. As such, the initial state can be regarded as a perturbation from which the controller needs to recover before entering the cyclic motion. Details on this initial behavior and, in general, the variability of the gait cycles are shown in detail in the Supplementary Material (Section 2). A well-chosen initial state minimizes the initial correction the controller must perform and helps to guide the optimization towards the intended target gait.

We do not optimize for specific motions through tracking objectives, but instead minimize a high-level cost function *J*, which consists of a velocity target and a term for minimizing muscle activation as a measure of muscle fatigue^70^:

3 $$J={{\mbox{w}}}_{{{{\rm{vel}}}}}\cdot {J}_{{{{\rm{vel}}}}}+{{\mbox{w}}}_{{{{\rm{act}}}}}\cdot {J}_{{{{\rm{act}}}}}$$

and mainly dictates the direction and speed of the gait. Another included optimization criterion is continuous gait without falling for the duration of the simulation *t*_sim_. Simulations are terminated after *t*_max_ = 30 s or if the center of mass (CoM) of the model goes below 0.5 m. A gait fulfills this criterion if it does not fall within the maximum simulation time, i.e., if *t*_sim_ = *t*_max_ This criterion is integrated into *J*_vel_.

To optimize for the five different gaits with the same workflow, we furthermore introduce a flag s_v_ into *J*_vel_, which allows to select between the different optimization conditions of reaching a target velocity *v*_tgt_ (flag *s*_v_ = 0) or maximizing the absolute velocity of the model (flag *s*_v_ = 1 or  −1 for forwards and backwards movements, respectively):

4 $${J}_{{{{\rm{vel}}}}}=\left\{\begin{array}{ll}\frac{d}{{t}_{{{{\rm{max}}}}}}\hfill\quad &\,{{{\rm{if}}}} \, {{{\rm{sv}}}}\,=-1\hfill\\ | 1-\frac{\bar{v}}{{v}_{{{{\rm{tgt}}}}}}| +1-\frac{{t}_{{{{\rm{sim}}}}}}{{t}_{{{{\rm{max}}}}}}\quad &\,{{{\rm{if}}}} \, {{{\rm{sv}}}}\,=0\hfill\\ -\frac{d}{{t}_{{{{\rm{max}}}}}}\hfill\quad &\,{{{\rm{if}}}} \, {{{\rm{sv}}}=1}\hfill\end{array}\right.{{{\rm{with}}}}\,\bar{v}=\frac{d}{\,{\mbox{max}}({t}_{{{{\rm{sim}}}}},2)}$$

Here, *d* is the completed distance, which corresponds to the horizontal displacement of the CoM of the segment closest to the origin at the end of the simulation (*t*_sim_). *J*_vel_ also encourages stability: if *s*_v_ ≠ 0, *J*_vel_ is calculated based on the maximum simulation time *t*_max_ (set to 30 s), penalizing early falls where with the same velocity the reached distance *d* is smaller (and thus *J*_vel_ is smaller) the earlier the model falls. If the optimization target is to reach a target velocity (*s*_v_ = 0), the average velocity of the model $\bar{v}$ has to be calculated based on the actual simulation time *t*_sim_ to be compared to the target velocity. To foster solutions that do not fall, a penalty for early terminations is added in this case ($1-\frac{{t}_{{{{\rm{sim}}}}}}{{t}_{{{{\rm{max}}}}}}$). Furthermore, we only calculate the velocity based on *t*_sim_ if *t*_sim_ > 2 s, as otherwise the optimization gets caught in local minima where the model falls within the first 2 s at a velocity close to the target velocity.

The muscle activation cost term *J*_act_ consists of integrated cubed activation during the time interval $[\frac{1}{3},\frac{2}{3}]{t}_{\max }$:

5 $${J}_{{{{\rm{act}}}}}=\frac{1}{d}\left(\int_{t = \frac{1}{3}{t}_{\max }}^{\frac{2}{3}{t}_{\max }}{\sum }_{i}{a}_{i}{(t)}^{3}\right).$$

Cubed muscle activation has been suggested as a measure of muscle fatigue^70,71^ based on experimental data indicating a cubic relationship between muscle force and endurance^72,73^. The restriction to the time interval $T=[\frac{1}{3},\frac{2}{3}]\,{t}_{\max }$ helps to initially encourage finding gaits without falls before minimizing fatigue ($t < \frac{1}{3}\,{t}_{\max }$) and prevents falling towards the end of the simulation due to micro-optimization of activations ($t > \frac{2}{3}\,{t}_{\max }$). As gains are constant throughout the simulation and the gaits emerge from the interplay of the reflexes, an adaptation to minimize the activation during $T=[\frac{1}{3},\frac{2}{3}]\,{t}_{\max }$ automatically influences the rest of the simulation as well. We do find that the gaits where activation minimization is active show some more inter-stride variability than the ones without; however, the strides are not different depending on which time interval they are in (compare Fig. S7a).

With the two cost function terms *J*_vel_ and *J*_act_ we then define the optimization parameters for the five target gaits. Humans typically walk at a preferred walking velocity, possibly minimizing energy expenditure^74^ and muscle fatigue^70^. The energetically optimal walking speed found by Ralston^74^ is at 1.23 m/s, and the subjects of the experimental dataset we use for comparison show a mean preferred speed of 1.17 m/s^38^. We therefore set a target velocity of 1.2 m/s for forwards walking. For simplicity, we set the same target velocity for backwards walking as well. Furthermore, we include activation minimization normalized to distance as a cost function term to take muscle fatigue into account^70^. For hopping and running, we optimize for maximum velocity and therefore deactivate the activation cost term. An overview of our optimization parameters can be found in Table 3. As discussed before, with this generic approach, the initial state has a large influence on the resulting gait, as e.g., hopping forward and running share the same optimization parameters and only differ in their initial state.

**Table 3:** Optimization parameters

Parameter	Walk Fwd	Walk Bkw	Hop Fwd	Hop Bkw	Run
w_vel_	100	100	100	100	100
–*v*_tgt_ (m/s)	1.2	-1.2	–	–	–
–s_v_	0	0	1	-1	1
w_act_	1000	1000	0	0	0

Chosen values of the optimization parameters for each of the five target gaits.

We optimize the parameters using the Covariance Matrix Adaptation Evolution Strategy (CMA-ES)^75^. The initial parameter mean values are rough estimates obtained through experimentation and are provided as part of the supplementary material. To allow sufficient exploration to cover all gaits and avoid local minima, we set the standard deviation to 0.2. We use the same mean values and standard deviations for all optimizations. Optimizations are terminated when the average relative improvement over the last 500 iterations is less than 10^−5^ per iteration.

Due to the complexity and discontinuity of our objective function (which involves a full musculoskeletal simulation in which the model can fall), our optimization problem is subject to many local minima. As a result, the optimization result highly depends on the stochastic sequence drawn by the CMA-ES optimizer. Due to these limitations, we run 20 optimizations for each target gait, each with different random seeds (which determine the stochastic sequence used by the optimizer), and pick the best result(s). While these results are unlikely to represent the absolute global optimum, we expect them to be close candidates.

### Experimental data

We compare the kinematics and muscular activations of our model to experimental data for walking and running. While humans are capable of performing hopping forward and backwards, unfortunately, there is a lack of experimental data for comparison.

The experimental data of human walking is mostly extracted from Camargo et al.^38^. We use the treadmill trials of all participants and extract ten strides at the velocity matching the walking velocity of 1.2 m/s of our model. For each participant, we then average over these strides. Next, we compute the mean and the mean absolute deviation from the mean and display this interval. To showcase the diversity in human gait, we also compare to data of one participant, who is closest to our model in terms of weight and height (AB06, 1.8 m, 74.8 kg). As Camargo et al.^38^ does not contain electromyography (EMG) data for ILI and BF, we furthermore extract data for these two muscles from other sources. ILI is compared to data taken from Perry^76^ as used in Geyer and Herr^28^ and BF data comes from Blazkiewicz^77^. As Perry^76^ and Blazkiewicz^77^ do not provide data of multiple subjects, we directly use the extracted data and do not display an interval for ILI and BF. Note, that the EMG data from Camargo et al.^38^ was normalized for each subject with respect to the average amplitude at a speed of 1.35 m/s, while data for BF from Blazkiewicz^77^ is given in μV and ILI data from Perry ^76^ was normalized to the maximum manual muscle test value. Therefore, a comparison of magnitude to our simulation data, normalized to maximum activation, is difficult.

Backward walking data was digitized from van Deursen et al.^78^ who normalized EMG data for each subject to the percent of maximal voluntary contraction. For running, we digitized data from Hamner and Delp^79^ of experienced long-distance runners running at 3 m/s. EMG normalization in Hamner and Delp^79^ was performed for each muscle using the maximum voltage across all trials for each subject.

To enable a quantitative comparison between simulation data and experimental data, we follow the approach of Geyer and Herr^28^ and compute the maximum cross-correlation *R* of the mean signal. We also extract the corresponding time shifts Δ expressed in percent of the gait cycle (maximum shift ${\Delta }_{\max }=20 \%$). Again, interpretation of the fit to EMG should be done carefully, as it has been argued that cross-correlation might not be suitable for comparing EMG values (between different subjects)^80^.

### Reporting summary

Further information on research design is available in the Nature Portfolio Reporting Summary linked to this article.

## Supplementary information

Supplementary material

Description of Additional Supplementary Files

Video S1 Target Gaits

Video S2 Within-Gait Variability

Video S3 Other Gaits

Video S4 Video Abstract

Reporting Summary

## Data Availability

The controller, model, and optimization results are available at 10.18419/DARUS-4176^81^. A video abstract is provided as supplementary video S4.

## References

[1] Allen, J. L. & Ting, L. H. Why is neuromechanical modeling of balance and locomotion so hard? In *Proc. Neuromechanical Modeling of Posture and Locomotion*, 197–223 (Springer, 2016).

[2] Kandel, E. R., Schwartz, J. H., Jessell, T. M., Siegelbaum, S. A. & Hudspeth, A. J. *Principles of Neural Science* 5th edn (McGraw-Hill, 2013).

[3] Sherrington, C. S. *The Integrative Action of The Nervous System* (Yale University Press, 1906).

[4] Sherrington, C. S. Flexion-reflex of the limb, crossed extension-reflex, and reflex stepping and standing. *J. Physiol.* **40**, 28–121 (1910). 16993027 10.1113/jphysiol.1910.sp001362 PMC1533734

[5] Levine, D. N. Sherrington’s the integrative action of the nervous system: a centennial appraisal. *J. Neurol. Sci.* **253**, 1–6 (2007). 17223135 10.1016/j.jns.2006.12.002

[6] Brown, T. G. The intrinsic factors in the act of progression in the mammal. *Proc. R. Soc. B* **84**, 308–319 (1911).

[7] Brown, T. G. On the nature of the fundamental activity of the nervous centres; together with an analysis of the conditioning of rhythmic activity in progression, and a theory of the evolution of function in the nervous system. *J. Physiol.* **48**, 18–46 (1914). 16993247 10.1113/jphysiol.1914.sp001646 PMC1420503

[8] Guertin, P. A. The mammalian central pattern generator for locomotion. *Brain Res. Rev.* **62**, 45–56 (2009). 19720083 10.1016/j.brainresrev.2009.08.002

[9] Kiehn, O. Decoding the organization of spinal circuits that control locomotion. *Nat. Rev. Neurosci.* **17**, 224–238 (2016). 26935168 10.1038/nrn.2016.9 PMC4844028

[10] Ijspeert, A. J. & Daley, M. A. Integration of feedforward and feedback control in the neuromechanics of vertebrate locomotion: a review of experimental, simulation and robotic studies. *J. Exp. Biol.* **226**, jeb245784 (2023). 10.1242/jeb.245784 37565347

[11] Tuthill, J. C. & Azim, E. Proprioception. *Curr. Biol.* **28**, R194–R203 (2018). 29510103 10.1016/j.cub.2018.01.064

[12] Ijspeert, A. J. Central pattern generators for locomotion control in animals and robots: a review. *Neural Netw.* **21**, 642–653 (2008). 18555958 10.1016/j.neunet.2008.03.014

[13] Côté, M.-P., Murray, L. M. & Knikou, M. Spinal control of locomotion: individual neurons, their circuits and functions. *Front. Physiol.* **9**, 784 (2018). 29988534 10.3389/fphys.2018.00784 PMC6026662

[14] Markin, S. N. et al. A neuromechanical model of spinal control of locomotion. In *Proc. Neuromechanical Modeling of Posture and Locomotion*, 21–65 (Springer, 2016).

[15] Rybak, I. A., Shevtsova, N. A., Markin, S. N., Prilutsky, B. I. & Frigon, A. Operation regimes of spinal circuits controlling locomotion and the role of supraspinal drives and sensory feedback. *Elife* **13**, RP98841 (2024). 39401073 10.7554/eLife.98841 PMC11473106

[16] Valero-Cuevas, F. J., Hoffmann, H., Kurse, M. U., Kutch, J. J. & Theodorou, E. A. Computational models for neuromuscular function. *IEEE Rev. Biomed. Eng.* **2**, 110–135 (2009). 21687779 10.1109/RBME.2009.2034981 PMC3116649

[17] Sartori, M., Llyod, D. G. & Farina, D. Neural data-driven musculoskeletal modeling for personalized neurorehabilitation technologies. *IEEE Trans. Biomed. Eng.* **63**, 879–893 (2016). 27046865 10.1109/TBME.2016.2538296

[18] Shourijeh, M. S., Mehrabi, N., McPhee, J. J. & Fregly, B. J. Advances in musculoskeletal modeling and their application to neurorehabilitation. *Front. Neurorobotics* **14**, 65 (2020). 10.3389/fnbot.2020.00065 PMC7581724 33162884

[19] Koelewijn, A. D. & Selinger, J. C. Predictive simulations to replicate human gait adaptations and energetics with exoskeletons. *IEEE Trans. Neural Syst. Rehabil. Eng.* **30**, 1931–1940 (2022). 35797329 10.1109/TNSRE.2022.3189038

[20] Wu, A. R. et al. An adaptive neuromuscular controller for assistive lower-limb exoskeletons: a preliminary study on subjects with spinal cord injury. *Front. Neurorobotics* **11**, 30 (2017). 10.3389/fnbot.2017.00030 PMC5476695 28676752

[21] Herr, H. M., Geyer, H. & Eilenberg, M. F. *Method for Using a Model-based Controller For a Robotic Leg* (Google Patents, 2019).

[22] Reinbolt, J. A., Haftka, R. T., Chmielewski, T. L. & Fregly, B. J. A computational framework to predict post-treatment outcome for gait-related disorders. *Med. Eng. Phys.* **30**, 434–443 (2008). 17616425 10.1016/j.medengphy.2007.05.005

[23] Eskinazi, I. & Fregly, B. J. A computational framework for simultaneous estimation of muscle and joint contact forces and body motion using optimization and surrogate modeling. *Med. Eng. Phys.* **54**, 56–64 (2018). 29487037 10.1016/j.medengphy.2018.02.002 PMC5864126

[24] Sauder, N. R. et al. Computational design of FastFES treatment to improve propulsive force symmetry during post-stroke gait: a feasibility study. *Front. Neurorobotics* **13**, 80 (2019). 10.3389/fnbot.2019.00080 PMC6779709 31632261

[25] Haeufle, D. F. B., Schmortte, B., Geyer, H., Müller, R. & Schmitt, S. The benefit of combining neuronal feedback and feed-forward control for robustness in step down perturbations of simulated human walking depends on the muscle function. *Front. Comput. Neurosci.* **12**, 80 (2018). 10.3389/fncom.2018.00080 PMC6190627 30356859

[26] Schreff, L., Haeufle, D. F., Vielemeyer, J. & Müller, R. Evaluating anticipatory control strategies for their capability to cope with step-down perturbations in computer simulations of human walking. *Sci. Rep.* **12**, 1–11 (2022). 35710689 10.1038/s41598-022-14040-0 PMC9203805

[27] Bunz, E. K., Haeufle, D. F., Remy, C. D. & Schmitt, S. Bioinspired preactivation reflex increases robustness of walking on rough terrain. *Sci. Rep.* **13**, 13219 (2023). 37580375 10.1038/s41598-023-39364-3 PMC10425464

[28] Geyer, H. & Herr, H. A muscle-reflex model that encodes principles of legged mechanics produces human walking dynamics and muscle activities. *IEEE Trans. on Neural Syst. Rehabil. Eng.* **18**, 263–273 (2010). 10.1109/TNSRE.2010.2047592 20378480

[29] Song, S. & Geyer, H. A neural circuitry that emphasizes spinal feedback generates diverse behaviours of human locomotion. *J. Physiol.* **593**, 3493–3511 (2015). 25920414 10.1113/JP270228 PMC4560581

[30] Dzeladini, F., Van Den Kieboom, J. & Ijspeert, A. The contribution of a central pattern generator in a reflex-based neuromuscular model. *Front. Human Neurosci.* **8**, 371 (2014). 10.3389/fnhum.2014.00371 PMC4071613 25018712

[31] Di Russo, A., Stanev, D., Armand, S. & Ijspeert, A. Sensory modulation of gait characteristics in human locomotion: a neuromusculoskeletal modeling study. *PLoS Comput. Biol.* **17**, e1008594 (2021). 34010288 10.1371/journal.pcbi.1008594 PMC8168850

[32] Ong, C. F., Geijtenbeek, T., Hicks, J. L. & Delp, S. L. Predicting gait adaptations due to ankle plantarflexor muscle weakness and contracture using physics-based musculoskeletal simulations. *PLoS Comput. Biol.* **15**, e1006993 (2019). 31589597 10.1371/journal.pcbi.1006993 PMC6797212

[33] Wang, J. M., Hamner, S. R., Delp, S. L. & Koltun, V. Optimizing locomotion controllers using biologically-based actuators and objectives. *ACM Trans. Graph.* **31**, 1–11 (2012). 10.1145/2185520.2185521 PMC4523558 26251560

[34] Milekovic, T. et al. A spinal cord neuroprosthesis for locomotor deficits due to parkinson’s disease. *Nat. Med.* **29**, 2854–2865 (2023). 37932548 10.1038/s41591-023-02584-1

[35] Veerkamp, K. et al. Evaluating cost function criteria in predicting healthy gait. *J. Biomech.* **123**, 110530 (2021). 34034014 10.1016/j.jbiomech.2021.110530

[36] Song, S. & Geyer, H. Evaluation of a neuromechanical walking control model using disturbance experiments. *Front. Comput. Neurosci.* **11**, 15 (2017). 28381996 10.3389/fncom.2017.00015 PMC5361655

[37] Ramadan, R., Geyer, H., Jeka, J., Schöner, G. & Reimann, H. A neuromuscular model of human locomotion combines spinal reflex circuits with voluntary movements. *Sci. Rep.* **12**, 8189 (2022). 35581211 10.1038/s41598-022-11102-1 PMC9114145

[38] Camargo, J., Ramanathan, A., Flanagan, W. & Young, A. A comprehensive, open-source dataset of lower limb biomechanics in multiple conditions of stairs, ramps, and level-ground ambulation and transitions. *J. Biomech.* **119**, 110320 (2021). 10.1016/j.jbiomech.2021.110320 33677231

[39] Prochazka, a, Gillard, D. & Bennett, D. J. Positive force feedback control of muscles. *J. Neurophysiol.* **77**, 3226–3236 (1997). 9212270 10.1152/jn.1997.77.6.3226

[40] Grey, M. J., Nielsen, J. B., Mazzaro, N. & Sinkjær, T. Positive force feedback in human walking. *J. Physiol.* **581**, 99–105 (2007). 17331984 10.1113/jphysiol.2007.130088 PMC2075215

[41] Winter, D. A. & Yack, H. EMG profiles during normal human walking: stride-to-stride and inter-subject variability. *Electroencephalogr. Clin. Neurophysiol.* **67**, 402–411 (1987). 2444408 10.1016/0013-4694(87)90003-4

[42] Pedotti, A. A study of motor coordination and neuromuscular activities in human locomotion. *Biol. Cybern.* **26**, 53–62 (1977). 861299 10.1007/BF00363992

[43] Song, S. & Geyer, H. Regulating speed in a neuromuscular human running model. In *Proc. IEEE-RAS 15th International Conference on Humanoid Robots (Humanoids)*, 217–222 (IEEE, 2015).

[44] Athletics, W. World records - men https://worldathletics.org/records/by-category/world-records (2024).

[45] Rathkey, J. K. & Wall-Scheffler, C. M. People choose to run at their optimal speed. *Am. J. Phys. Anthropol.* **163**, 85–93 (2017). 28195301 10.1002/ajpa.23187

[46] Brooks, L. C., Weyand, P. G. & Clark, K. P. Does restricting arm motion compromise short sprint running performance? *Gait Posture* **94**, 114–118 (2022). 35276457 10.1016/j.gaitpost.2022.03.001

[47] Rybak, I. A., Dougherty, K. J. & Shevtsova, N. A. Organization of the mammalian locomotor CPG: review of computational model and circuit architectures based on genetically identified spinal interneurons. *ENeuro* **2**, ENEURO.0069-15.2015 (2015). 10.1523/ENEURO.0069-15.2015 PMC4603253 26478909

[48] Loeb, G. E. The control and responses of mammalian muscle spindles during normally executed motor tasks. *Exerc. Sport Sci. Rev.* **12**, 157–204 (1984). 6234174

[49] Prochazka, A. & Gorassini, M. Ensemble firing of muscle afferents recorded during normal locomotion in cats. *J. Physiol.* **507**, 293–304 (1998). 9490855 10.1111/j.1469-7793.1998.293bu.x PMC2230769

[50] Niyo, G., Almofeez, L. I., Erwin, A. & Valero-Cuevas, F. J. A computational study of how an *α*-to *γ*-motoneurone collateral can mitigate velocity-dependent stretch reflexes during voluntary movement. *Proc. Natl. Acad. Sci. USA* **121**, e2321659121 (2024). 39116178 10.1073/pnas.2321659121 PMC11348295

[51] Valero-Cuevas, F. J. *Fundamentals of Neuromechanics*, vol. 8 (Springer, 2016).

[52] Büschges, A. & El Manira, A. Sensory pathways and their modulation in the control of locomotion. *Curr. Opin. Neurobiol.* **8**, 733–739 (1998). 9914236 10.1016/s0959-4388(98)80115-3

[53] Messara, S., Manzoori, A. R., Di Russo, A., Ijspeert, A. & Bouri, M. Novel design and implementation of a neuromuscular controller on a hip exoskeleton for partial gait assistance. In Proc. 2023 *International Conference on Rehabilitation Robotics (ICORR)*, 1–6 (IEEE, 2023). 10.1109/ICORR58425.2023.10304758 37941265

[54] Millard, M., Uchida, T., Seth, A. & Delp, S. L. Flexing computational muscle: modeling and simulation of musculotendon dynamics. *J. Biomech. Eng.* **135**, 021005 (2013). 23445050 10.1115/1.4023390 PMC3705831

[55] Delp, S. L. et al. An interactive graphics-based model of the lower extremity to study orthopaedic surgical procedures. *IEEE Trans. Biomed. Eng.* **37**, 757–767 (1990). 2210784 10.1109/10.102791

[56] Arnold, E. M., Ward, S. R., Lieber, R. L. & Delp, S. L. A model of the lower limb for analysis of human movement. *Ann. Biomed. Eng.* **38**, 269–279 (2010). 19957039 10.1007/s10439-009-9852-5 PMC2903973

[57] Rajagopal, A. et al. Full-body musculoskeletal model for muscle-driven simulation of human gait. *IEEE Trans. Biomed. Eng.* **63**, 2068–2079 (2016). 27392337 10.1109/TBME.2016.2586891 PMC5507211

[58] Le Sant, G. et al. Stiffness mapping of lower leg muscles during passive dorsiflexion. *J. Anat.* **230**, 639–650 (2017). 28251615 10.1111/joa.12589 PMC5382595

[59] Zajac, F. E. Muscle and tendon: properties, models, scaling, and application to biomechanics and motor control. *Crit. Rev. Biomed. Eng.* **17**, 359–411 (1989). 2676342

[60] Hunt, K. H. & Crossley, F. R. E. Coefficient of restitution interpreted as damping in vibroimpact. *J. Appl. Mech.* **42**, 440–445 (1975).

[61] Seth, A. et al. OpenSim: simulating musculoskeletal dynamics and neuromuscular control to study human and animal movement. *PLoS Comput. Biol.* **14**, e1006223 (2018). 10.1371/journal.pcbi.1006223 PMC6061994 30048444

[62] Geijtenbeek, T. Scone: open source software for predictive simulation of biological motion. *J. Open Source Softw.* **4**, 1421 (2019).

[63] Geijtenbeek, T. The Hyfydy simulation software https://hyfydy.com. (2021).

[64] Oliver, K. M. et al. Molecular correlates of muscle spindle and Golgi tendon organ afferents. *Nat. Commun.* **12**, 1451 (2021). 33649316 10.1038/s41467-021-21880-3 PMC7977083

[65] Abbott, E. M. et al. Attenuation of muscle spindle firing with artificially increased series compliance during stretch of relaxed muscle. *Exp. Physiol.* **109**, 148–158 (2024). 37856330 10.1113/EP090872 PMC10841431

[66] Blum, K. P., Lamotte D’Incamps, B., Zytnicki, D. & Ting, L. H. Force encoding in muscle spindles during stretch of passive muscle. *PLoS Comput. Biol.* **13**, e1005767 (2017). 28945740 10.1371/journal.pcbi.1005767 PMC5634630

[67] Jankowska, E. Interneuronal relay in spinal pathways from proprioceptors. *Prog Neurobiol.* **38**, 335–78 (1992). 10.1016/0301-0082(92)90024-9 1315446

[68] Pearson, K. G. & Collins, D. F. Reversal of the influence of group Ib afferents from plantaris on activity in medial gastrocnemius muscle during locomotor activity. *J. Neurophysiol.* **70**, 1009–1017 (1993). 8229157 10.1152/jn.1993.70.3.1009

[69] van der Kruk, E. & Geijtenbeek, T. A planar neuromuscular controller to simulate compensation strategies in the sit-to-walk movement. *PLoS ONE* **19**, e0305328 (2024). 38870249 10.1371/journal.pone.0305328 PMC11175457

[70] Ackermann, M. & Van den Bogert, A. J. Optimality principles for model-based prediction of human gait. *J. Biomech.* **43**, 1055–1060 (2010). 20074736 10.1016/j.jbiomech.2009.12.012 PMC2849893

[71] Johnson, R. T., Bianco, N. A. & Finley, J. M. Patterns of asymmetry and energy cost generated from predictive simulations of hemiparetic gait. *PLoS Comput. Biol.* **18**, e1010466 (2022). 36084139 10.1371/journal.pcbi.1010466 PMC9491609

[72] Crowninshield, R. D. & Brand, R. A. A physiologically based criterion of muscle force prediction in locomotion. *J. Biomech.* **14**, 793–801 (1981). 7334039 10.1016/0021-9290(81)90035-x

[73] Miller, R. H., Umberger, B. R., Hamill, J. & Caldwell, G. E. Evaluation of the minimum energy hypothesis and other potential optimality criteria for human running. *Proc. R. Soc. B Biol. Sci.* **279**, 1498–1505 (2012). 10.1098/rspb.2011.2015 PMC3282349 22072601

[74] Ralston, H. J. Energy-speed relation and optimal speed during level walking. *Int. Z. Angew. Physiol. Einschl. Arbeitsphysiol.* **17**, 277–283 (1958). 10.1007/BF00698754 13610523

[75] Hansen, N., Müller, S. D. & Koumoutsakos, P. Reducing the time complexity of the derandomized evolution strategy with covariance matrix adaptation (CMA-ES). *Evolut. Comput.* **11**, 1–18 (2003). 10.1162/106365603321828970 12804094

[76] Perry, J. *Gait Analysis. Normal and Pathological Function* (SLACK Inc., 1992).

[77] Błażkiewicz, M. Muscle force distribution during forward and backward locomotion. *Acta Bioeng. Biomech.* **15**, 3–9 (2013). 24215105

[78] van Deursen, R. W., Flynn, T. W., McCrory, J. L. & Morag, E. Does a single control mechanism exist for both forward and backward walking? *Gait Posture* **7**, 214–224 (1998). 10200387 10.1016/s0966-6362(98)00007-1

[79] Hamner, S. R. & Delp, S. L. Muscle contributions to fore-aft and vertical body mass center accelerations over a range of running speeds. *J. Biomech.* **46**, 780–787 (2013). 23246045 10.1016/j.jbiomech.2012.11.024 PMC3979434

[80] Wren, T. A., Do, K. P., Rethlefsen, S. A. & Healy, B. Cross-correlation as a method for comparing dynamic electromyography signals during gait. *J. Biomech.* **39**, 2714–2718 (2006). 16219314 10.1016/j.jbiomech.2005.09.006

[81] Bunz, E. K., Haeufle, D. F., Schmitt, S. & Geijtenbeek, T. Repository for “A spinal network of proprioceptive reflexes can produce a variety of bipedal gaits” 10.18419/DARUS-4176. (2025). 10.1038/s42003-025-09307-x PMC12783750 41402529

