# Supplementary material for: A spinal network of proprioceptive reflexes can produce a variety of bipedal gaits
Source: Commun Biol. 2025 Dec 16;9:36. doi: 10.1038/s42003-025-09307-x (PMC12783750; doi:10.1038/s42003-025-09307-x)
Supplement: Supplementary file 1 — Supplementary material [file 42003_2025_9307_MOESM1_ESM.pdf]

Supplementary Material for *A spinal network of proprioceptive reflexes can produce a variety of bipedal gaits*

Elsa K. Bunz*, Daniel F. B. Haeufle, Syn Schmitt, Thomas Geijtenbeek

* elsa.bunz@imsb.uni-stuttgart.de

1 Model

Table S 1. Model parameters. Optimal fiber length l_{opt}, tendon slack length l_{slack}, maximum isometric force F_{max}, pennation at optimal fiber length α (taken from [1], with exception of hamstring parameters which were taken from [2]), muscle grouping and modelled delays Δt (taken from [3], homonymous and antagonistic reflex, for antagonistic reflexes the mean of both involved muscles is used) of the 9 Hill-type muscles per leg. For more details see Section "Model" of main publication.

	l_{opt}(cm)	l_{slack}(cm)	F_{max} (N)	α (°)	Muscles	Δt (ms) Hom.	Ant.
GLU	15.69	11.1	1944	21.9	Gluteus max. super. + middle + infer.	10	10
ILI	10.66	15.2	2186	14.3	Psoas	10	10
RF	7.59	34.49	1169	13.9	Rectus fem.	20	20
HAM	9.76	31.9	2594	11.6	Biceps fem. l. head, semimem. semiten.	15	20
BF	11.03	9.5	804	12.3	Biceps fem. s. head	20	20
VAS	9.93	12.31	4530	4.5	Vastus intermedius, lateralis, medialis	20	20
GAS	5.1	38.4	2241	9.9	Gastrocnemius lateral + medial head	35	35
TA	6.83	24.3	1579	9.6	Tibialis anterior	35	35
SOL	4.4	24.8	3549	28.3	Soleus	35	35

2 Initial state

Table S 2. Initial model state. Initial state values ϕ (rad or m) and velocities $\dot{\phi}$ (rad/s or m/s) of the model for all five target gaits, manually tuned and obtained from the gait cycle of initially found solutions for the movement.

	Walk Fwd		Walk Bkw		Run		Hop Fwd		Hop Bkw	
	ϕ	$\dot{\phi}$	ϕ	$\dot{\phi}$	ϕ	$\dot{\phi}$	ϕ	$\dot{\phi}$	ϕ	$\dot{\phi}$
pelvis_tilt	-0.03	-0.57	-0.1	-0.05	-0.08	1.39	-0.4	1.68	-0.3	0
pelvis_tx	0	1	0	-0.8	0	4.49	0	1.55	0	-0.5
pelvis_ty	0.95	0.05	0.95	-0.03	0.94	-0.63	0.91	0.72	0.8	0
hip_flexion_r	0.17	-0.58	0.1	-0.3	-0.19	2.11	0.6	-6.74	0.7	0
knee_angle_r	-0.1	-1	-0.1	-0.05	-0.85	-9.72	-0.36	5.77	-1	0
ankle_angle_r	0.02	0.99	0.2	0.1	-0.78	0.46	0.16	-1.9	0.5	0
hip_flexion_l	0.23	4.91	0.7	-3	0.24	-6.87	0.6	-6.74	0.7	0
knee_angle_l	-1.23	-8.6	-1.1	-0.3	0.06	-0.03	-0.36	5.77	-1	0
ankle_angle_l	-0.17	0.63	-0.2	-0.1	-0.11	7.4	0.16	-1.9	0.5	0

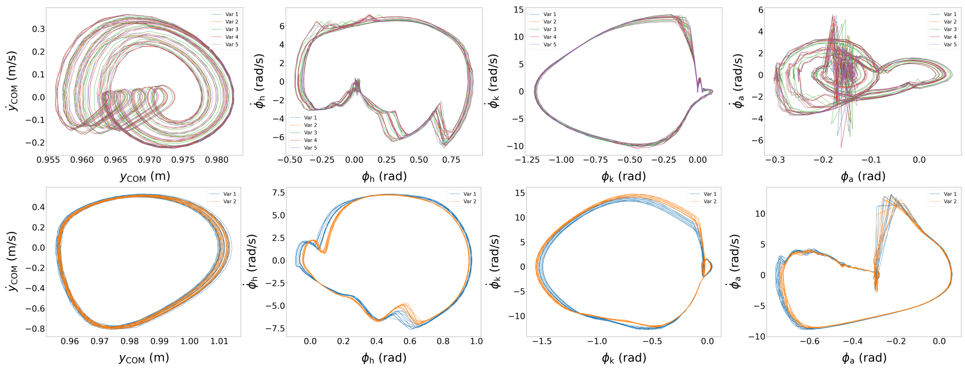

Fig S 1. Phase plots for small, random variations of the initial states (1%) which lead to stable movement. Top: walking, Bottom: running

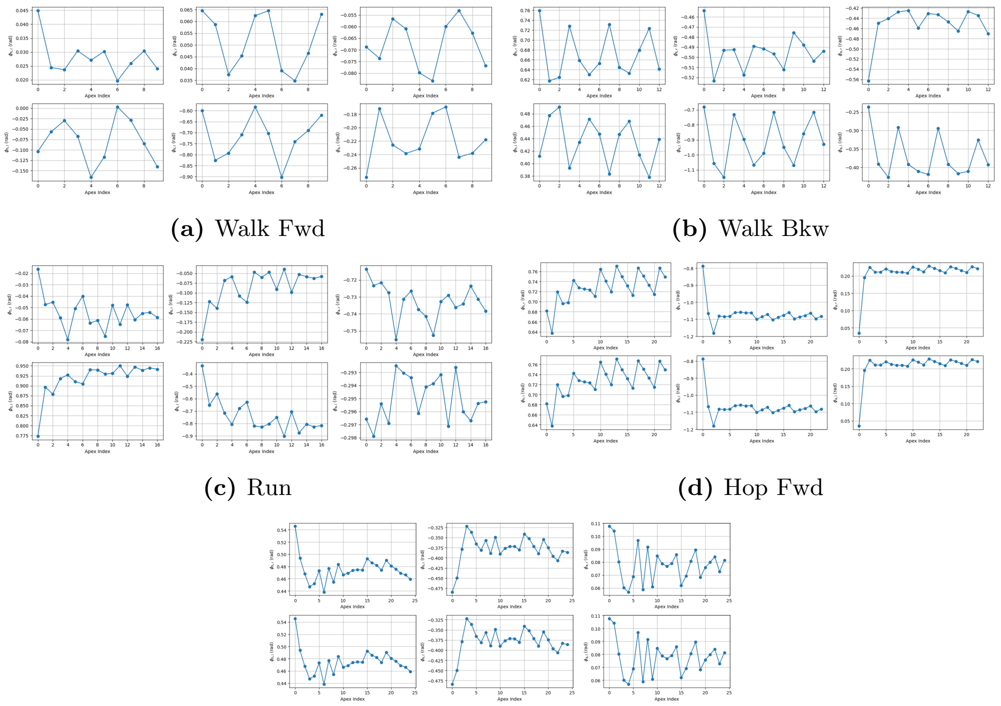

(a) Walk Fwd **(b)** Walk Bkw

(c) Run **(d)** Hop Fwd

(e) Hop Bkw

Fig S 2. Joint angles of hip, knee and ankle joint at COM Apex for the first 10 s for all five target gaits. During rhythmic movement the initial states for each gait cycle show marked variations. For walking and running only every second COM apex is displayed as during one gait cycle two COM apexes happen.

3 Target gaits

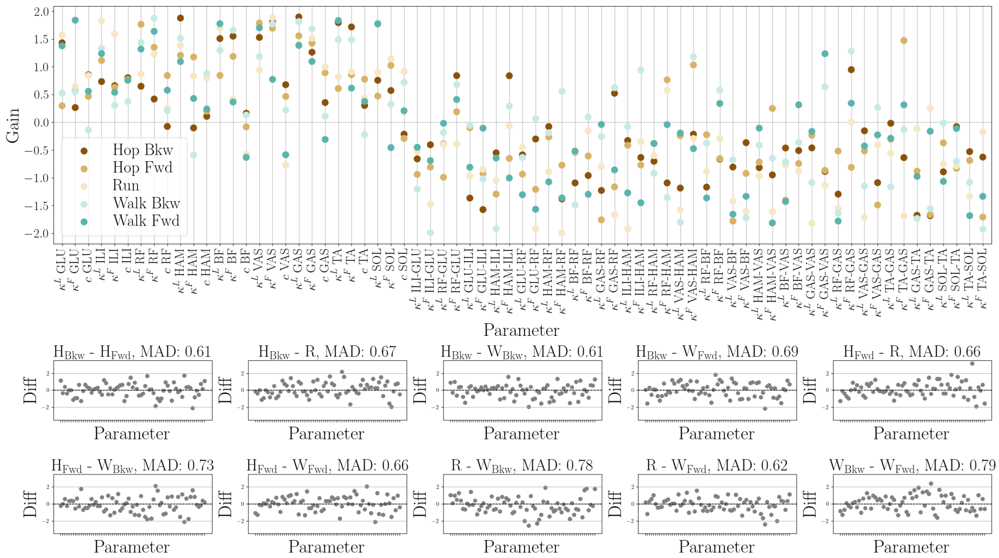

Fig S 3. Comparison of the resulting parameter sets for the five target gaits. MAD: mean average deviation. W: Walk, H: Hop, R: Run

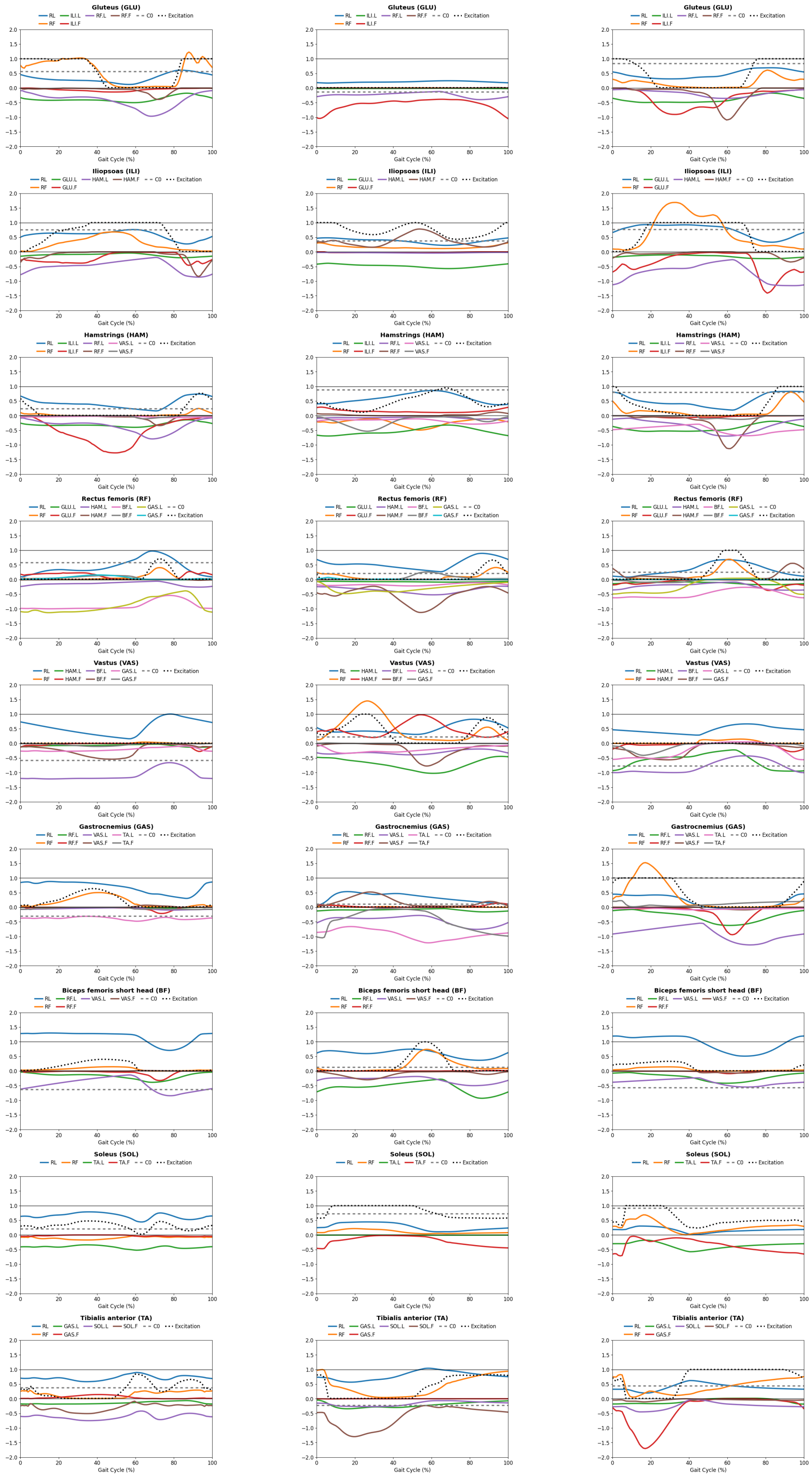

Fig S 4. Time profile of the reflex contributions for each muscle and the resulting excitation for Walk fwd (left), Walk bkw (center), Run (left). RL and RF represent the monosynaptic reflex using the feedback coming from the target muscle and MUSCLE.RL/RF shows reflexes based on the feedback from source muscle MUSCLE.

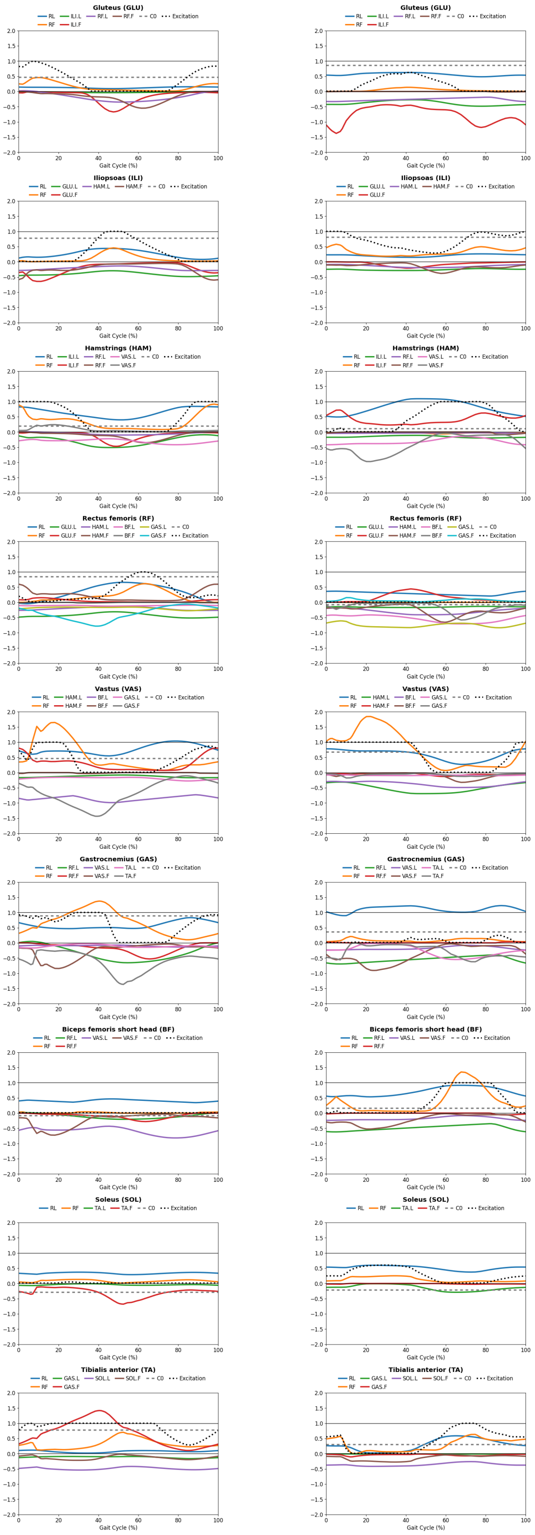

Fig S 5. Time profile of the reflex contributions for each muscle and the resulting excitation for Hop fwd (left) and Hop bkw (right). RL and RF represent the monosynaptic reflex using the feedback coming from the target muscle and MUSCLE.RL/RF shows reflexes based on the feedback from source muscle MUSCLE.

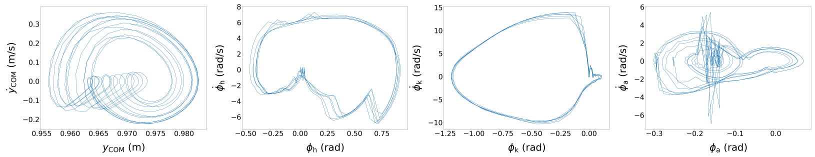

(a) Walk Fwd

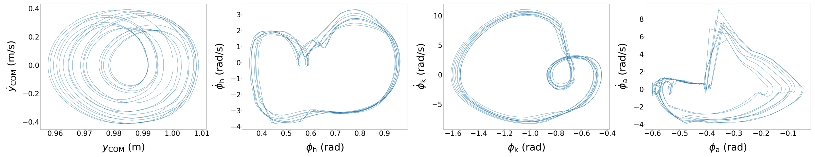

(b) Walk Bkw

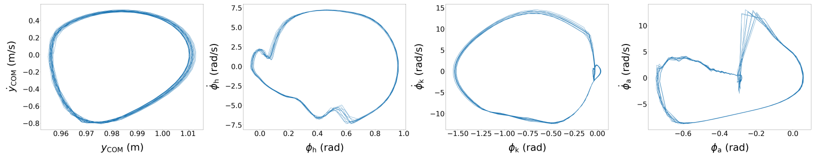

(c) Run

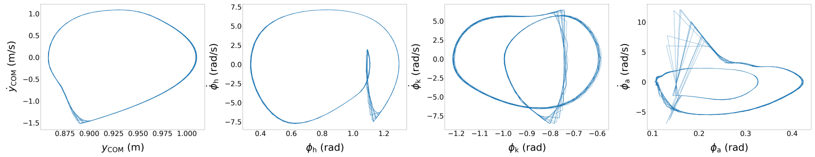

(d) Hop Fwd

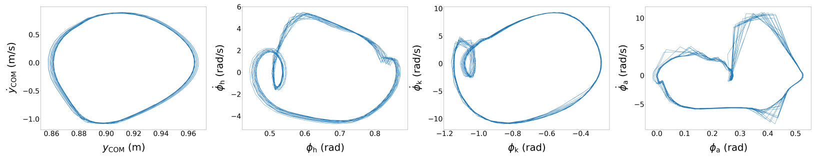

(e) Hop Bkw

Fig S 6. Phase plots of the five target gaits for center of mass (COM) height, and hip, knee and ankle angles (left to right).

Walk Fwd

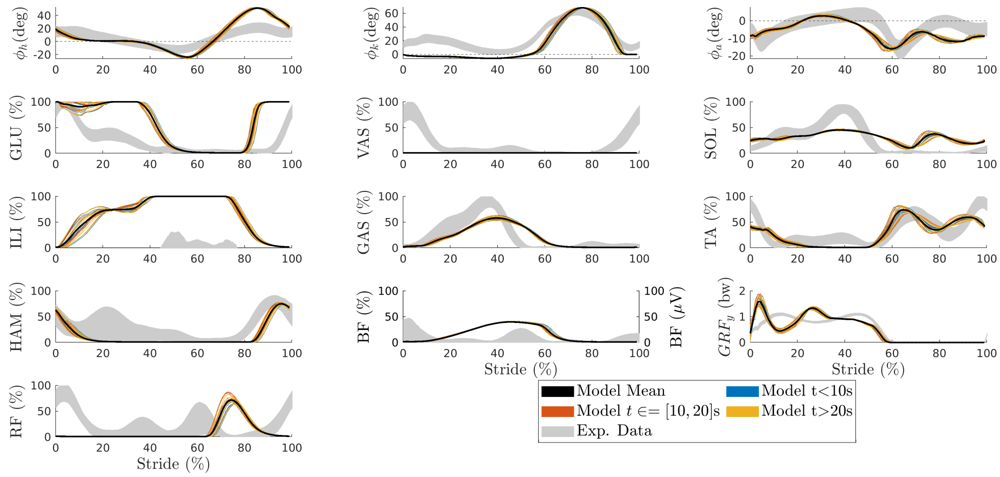

(a) Walk Fwd ($1.2\,\mathrm{m\,s^{-1}}$)

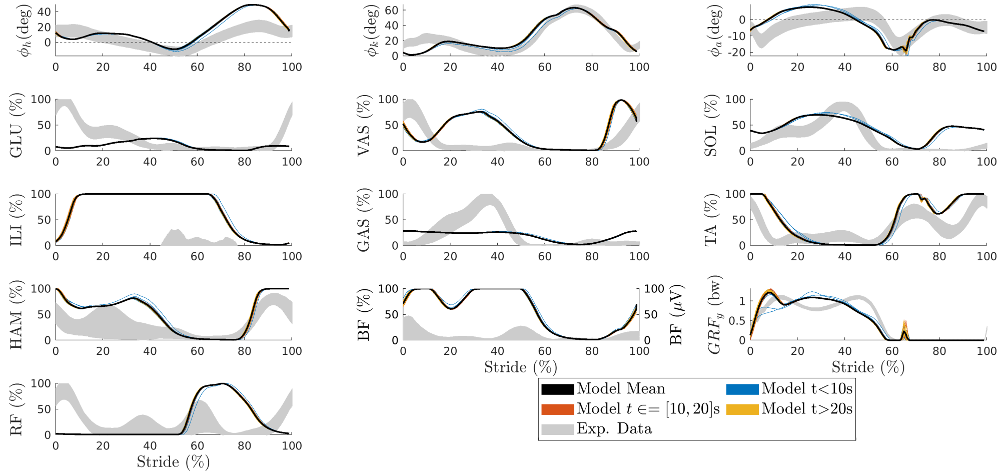

(b) Walk Fwd 2 ($1.1\,\mathrm{m\,s^{-1}}$)

Fig S 7. Kinematics, GRF and muscular activation Joint angles and muscular activations for hip, knee and ankle joint (left to right) as well as ground reaction force for (a) our target gait Walk Fwd and for (b) a different parameter set Walk Fwd 2 generating forwards walking with knee flexion during stance and VAS activation. Strides are colored based on the time interval they start in ($t < 10\,\mathrm{s}$, $t \in 10\,\mathrm{s}$ to $20\,\mathrm{s}$, $t > 20\,\mathrm{s}$) and maximum simulation time is set to $t_{\max} = 30\,\mathrm{s}$.

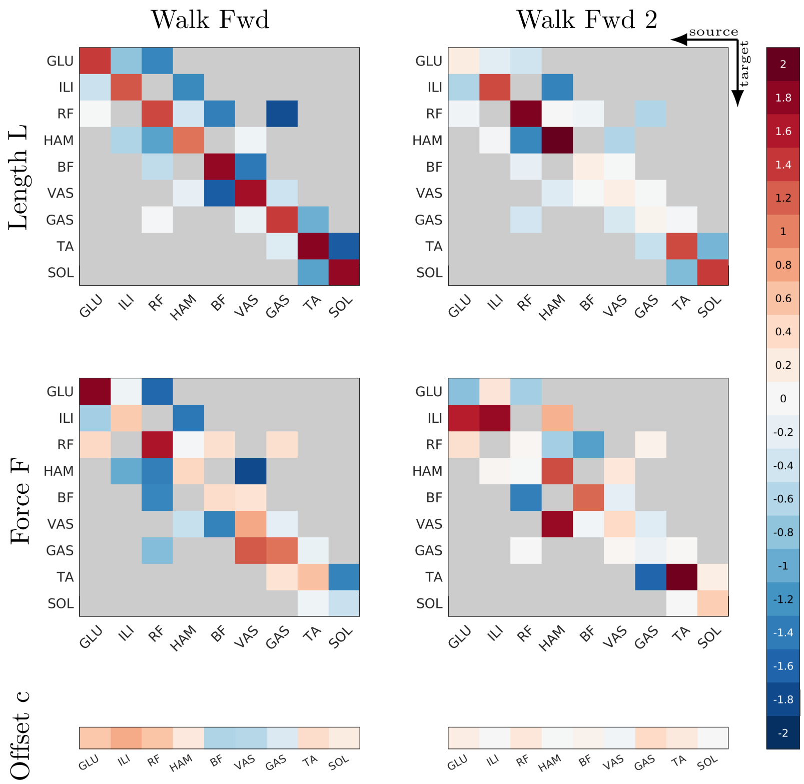

Fig S 8. Parameter sets Walk Fwd Comparison of two parameters sets generating forwards walking: Walk Fwd (left) the found target gait and Walk Fwd 2 (right) that was manually found and exhibits more knee flexion during stance. Matrix columns represent the source muscle the feedback is coming from, rows show the target muscle receiving a stimulation input from the reflex connection.

Table S 3. Maximum cross-correlation R and time shift Δ (%) for muscular activation Comparison of mean of experimental data from [4–6] (exp) or mean data of subject AB06 from [4] and simulation data from our model (our) or the model of Geyer & Herr [7] (GH) as well as the second forwards walking solution Walk Fwd 2 (our,2).

	Glu		Vas		Sol		Ili		Gas	
	R	Δ	R	Δ	R	Δ	R	Δ	R	Δ
exp-our	0.81	0	0.58	0	0.82	1.8	0.69	3.7	0.97	7.4
exp-GH	0.86	10.4	0.74	9.7	0.99	8.8	0.88	4.7	0.99	11.2
exp-our,2	0.72	20	0.55	1.2	0.85	1.1	0.64	13.5	0.73	0
AB06-our	0.83	0	0.91	0	0.83	0.6	–	–	0.97	11.5
AB06-GH	0.83	10.8	0.65	8.8	0.98	9.7	–	–	0.94	14.3

	Ta		Ham		Bf		Rf	
	R	Δ	R	Δ	R	Δ	R	Δ
exp-our	0.91	6.0	0.83	0	0.55	9	0.37	14
exp-GH	0.82	0	0.55	0	–	–	–	–
exp-our,2	0.93	0	0.94	0	0.8	0	0.33	8.8
S06-our	0.94	0.3	0.86	1.2	–	–	–	–
S06-GH	0.83	0	0.35	6.4	–	–	–	–

Table S 4. Maximum cross-correlation R for joint angles and GRF Similarity metrics computed between mean of experimental walking data (exp) and simulation data of the second forwards walking solution of our model (our,2) in comparison to the results of our target gait Walk Fwd (our) and the model of [7] (GH) already given in the main results Table 3.

	ϕ_h		ϕ_k		ϕ_a		GRF	
	R	Δ	R	Δ	R	Δ	R	Δ
exp-our,2	0.84	1.3	0.97	0.7	0.76	3.9	0.97	2.3
exp-our	0.90	0	0.89	2.1	0.79	3.4	0.93	3.6
exp -GH	0.93	0	0.98	0.6	0.69	1	0.99	0.7

Run

Table S 5. Maximum cross-correlation R and time shift Δ (%) for muscular activation during Run Computed between mean of experimental data (exp) from [8] and simulation data from our model (our).

	Glu		Vas		Sol		Ili		Gas	
	R	Δ	R	Δ	R	Δ	R	Δ	R	Δ
exp-our	0.89	0	0.56	0	0.82	8.6	–	–	0.91	2.8

	Ta		Ham		Bf		Rf	
	R	Δ	R	Δ	R	Δ	R	Δ
exp-our	0.95	0	0.85	0	–	–	0.56	7.7

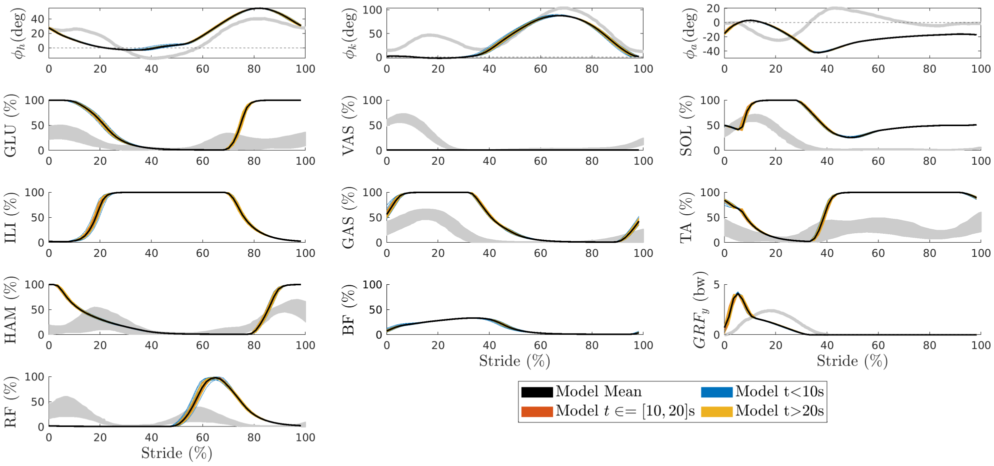

Fig S 9. Kinematics, GRF and muscular activation Joint angles and muscular activations for hip, knee and ankle joint (left to right) as well as ground reaction force for Run. Strides are colored based on the time interval they start in ($t < 10\,\text{s}$, $t \in 10\,\text{s}$ to $20\,\text{s}$, $t > 20\,\text{s}$) and maximum simulation time is set to $t_{\max} = 30\,\text{s}$.

Other target gaits

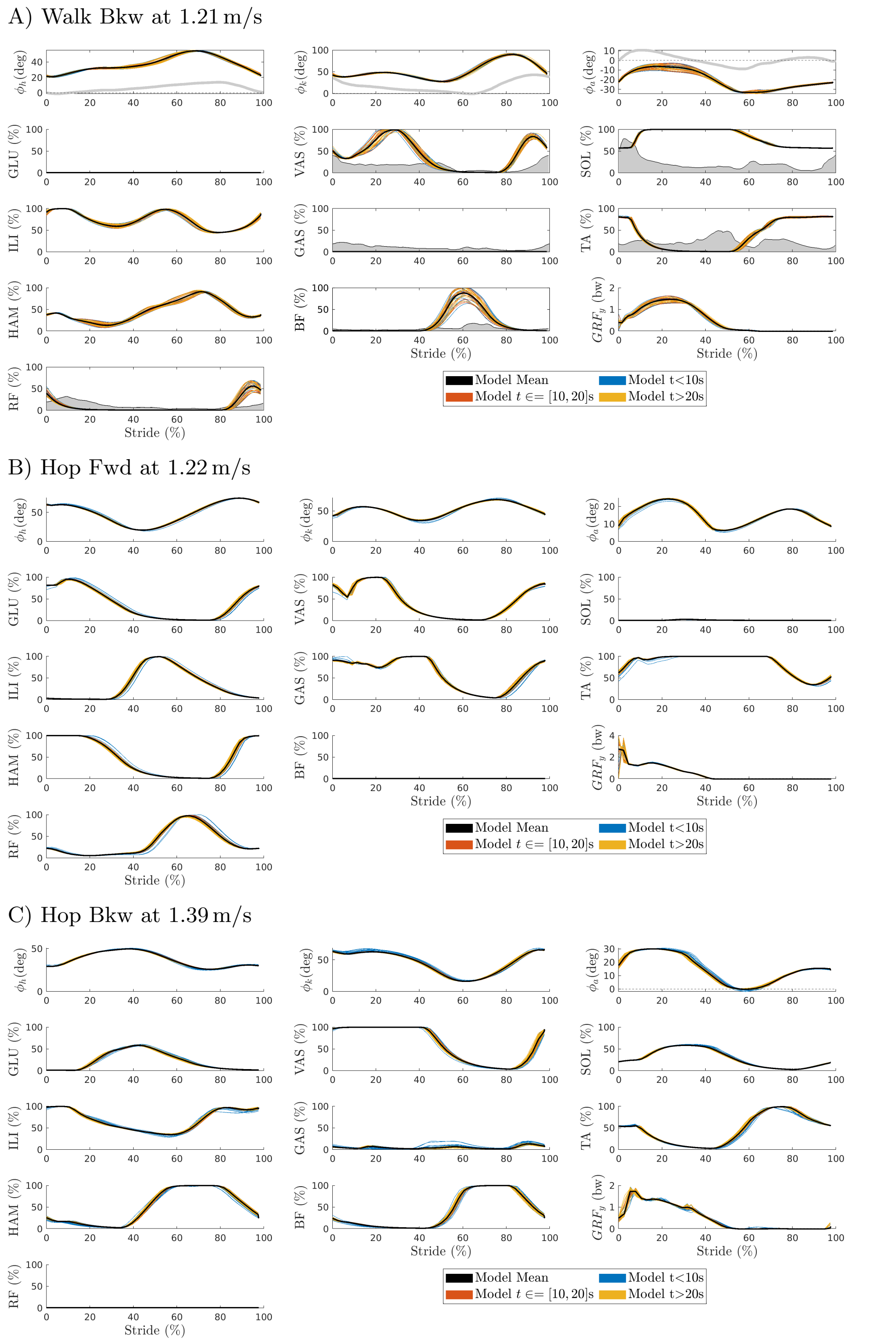

Fig S 10. Kinematics, GRF and muscular activation Joint angles and muscular activations for hip, knee and ankle joint (left to right) as well as ground reaction force. Strides are colored based on the time interval they start in ($t < 10$ s, $t \in 10$ s to 20 s, $t > 20$ s) and maximum simulation time is set to $t_{\max} = 30$ s.

Table S 6. Maximum cross-correlation R for joint angles and GRF Similarity metrics computed between mean of experimental data of backward walking from [9] (exp) and simulation data of our model (our).

	ϕ_h		ϕ_k		ϕ_a		GRF	
	R	Δ	R	Δ	R	Δ	R	Δ
exp-our	0.94	4.7	0.85	1.7	0.25	11.3	–	–

Table S 7. Maximum cross-correlation R and time shift Δ (%) for muscular activation during backard walking Computed between mean of experimental data (exp) from [9] and simulation data from our model (our).

	Glu		Vas		Sol		Ili		Gas	
	R	Δ	R	Δ	R	Δ	R	Δ	R	Δ
exp-our	–	–	0.83	0.8	0.77	8.4	–	–	0.9	0

	Ta		Ham		Bf		Rf	
	R	Δ	R	Δ	R	Δ	R	Δ
exp-our	0.65	20.0	–	–	0.88	5.6	0.52	0

References

1. Delp SL, Loan JP, Hoy MG, Zajac FE, Topp EL, Rosen JM. An interactive graphics-based model of the lower extremity to study orthopaedic surgical procedures. IEEE Transactions on Biomedical engineering. 1990;37(8):757–767. doi:10.1109/10.102791.

2. Arnold EM, Ward SR, Lieber RL, Delp SL. A model of the lower limb for analysis of human movement. Annals of biomedical engineering. 2010;38:269–279. doi:10.1007/s10439-009-9852-5.

3. van der Kruk E, Geijtenbeek T. A planar neuromuscular controller to simulate compensation strategies in the sit-to-walk movement. PLoS one. 2024;19(6):e0305328.

4. Camargo J, Ramanathan A, Flanagan W, Young A. A comprehensive, open-source dataset of lower limb biomechanics in multiple conditions of stairs, ramps, and level-ground ambulation and transitions. Journal of Biomechanics. 2021; p. 110320.

5. Perry J. Gait analysis. Normal and pathological function. Thorofare, NJ: SLACK Inc; 1992.

6. Błażkiewicz M. Muscle force distribution during forward and backward locomotion. Acta of Bioengineering and Biomechanics. 2013;15(3):3–9.

7. Geyer H, Herr H. A muscle-reflex model that encodes principles of legged mechanics produces human walking dynamics and muscle activities. IEEE Transactions on Neural Systems and Rehabilitation Engineering. 2010; p. 263–273.

8. Hamner SR, Delp SL. Muscle contributions to fore-aft and vertical body mass center accelerations over a range of running speeds. Journal of biomechanics. 2013;46(4):780–787.

9. van Deursen RW, Flynn TW, McCrory JL, Morag E. Does a single control mechanism exist for both forward and backward walking? Gait & Posture. 1998;7(3):214–224.
