# Supplementary material for: A spinal network of proprioceptive reflexes can produce a variety of bipedal gaits
Source: Commun Biol. 2025 Dec 16;9:36. doi: 10.1038/s42003-025-09307-x (PMC12783750; doi:10.1038/s42003-025-09307-x)
Supplement: Supplementary file 2 — Description of Additional Supplementary Files [file 42003_2025_9307_MOESM2_ESM.docx]

**Description of Additional Supplementary Files

File name:** Video S1 **Description:** Video showing the results for the five target gaits walking forwards and backwards, hopping forwards and backwards and running. **File name:** Video S2 **Description:** Video showing the variations the controller can generate within each of the five target gaits.
 **File name:** Video S3 **Description:** Video showing other interesting gaits the controller can generate that do not fit within the target gaits but showcase the versatility. **File name:** Video S4 **Description:** Video Abstract summarizing the main results of the paper.
